# Satisfaction of Patients with Multiple Sclerosis Towards the Provision of Healthcare Services at a Tertiary Care Setting in Saudi Arabia

**DOI:** 10.3390/clinpract14060217

**Published:** 2024-12-20

**Authors:** Dalal Salem Al-Dossari, Ibrahim Abdulaziz Al-Zaagi, Mashael Abdulrahman Bin Salamma, Faisal Abdulaziz Alghamdi, Ahmed Hasan Althobaiti, Abdullah Bin Dakhil Allah bin Awad Al-Harbi, Abdullah Rashed Alshelali, Anham Nabil Hassan, Baleegh Mohammed Ali, Sheraz Ali

**Affiliations:** 1 Pharmaceutical Care Services, King Saud Medical City, Ministry of Health, Riyadh 12746, Saudi Arabia; d.dawsari@ksmc.med.sa (D.S.A.-D.); izaagi@ksmc.med.sa (I.A.A.-Z.); misha.ksmc@gmail.com (M.A.B.S.); ph_faisal@hotmail.com (F.A.A.); 2 King Saud Medical City, Ministry of Health, Riyadh 12746, Saudi Arabia; ah.althobaiti@ksmc.med.sa (A.H.A.); ajfn2015@hotmail.com (A.B.D.A.b.A.A.-H.); ars-1@live.com (A.R.A.); a.sayed@ksmc.med.sa (A.N.H.); dr.baleegh@gmail.com (B.M.A.); 3 Centre for Clinical Research, Department of Health, Tasmanian Government, Hobart 7000, Australia; 4 College of Health and Medicine, University of Tasmania, Hobart 7000, Australia

**Keywords:** multiple sclerosis, satisfaction, CANHELP, factors

## Abstract

Background: Multiple sclerosis (MS) is an autoimmune-mediated neurological disorder and the most frequent neurological disability in young adults. Assessing MS patient satisfaction with care is necessary to evaluate healthcare service quality and factors impacting it. Objectives: We aimed to determine the satisfaction of patients with MS towards the provision of healthcare services, and the factors affecting their satisfaction. Methods: We conducted a cross-sectional survey from November 2022 to December 2022 at King Saud Medical City, Saudi Arabia. The study participants completed the CANHELP Lite27 questionnaire, which consists of 21 items. Results: A total of 300 patients with MS participated in this study. The majority were female (80%), with an average age of 31.6 years and a mean disease duration of 5.22 years. Overall, satisfaction was moderate (63.59 ± 14.54). Patients were most satisfied with general aspects of care (72.00 ± 16.46), doctor relationships (68.58 ± 14.88), and communication (67.72 ± 17.60), but less so with decision-making (64.98 ± 18.37) and illness management (59.60 ± 16.31). Correlation analysis revealed a negative association between disease duration and satisfaction across all domains (correlation coefficient ranged from −0.290 to −0.206, *p* < 0.01), while age showed no significant correlation (*p* > 0.05). Multiple linear regression identified age as positively influencing satisfaction, while longer disease duration had a negative impact. Higher education and marital status were associated with increased satisfaction, while employment status and having children showed mixed results. Conclusions: This research uncovered significant insights regarding MS patient satisfaction within healthcare services. Despite moderate satisfaction levels overall, specific interventions are necessary to address shortcomings in decision-making and illness management. The negative correlation between disease duration and satisfaction across all domains underscores the evolving needs of MS patients over time. Future research could examine the effectiveness of illness management programs in improving MS patient satisfaction.

## 1. Introduction

Multiple sclerosis is an autoimmune-mediated neurological disorder and the most frequent neurological disability in young people [1]. Multiple sclerosis is an inflammatory condition that tends to trigger cognitive, emotional, psychological, and social functioning problems [2]. Patients with multiple sclerosis often have complicated needs, necessitating diverse health services [3]. This disease affects 2.5 million people, and the global prevalence is 33/100,000 [4,5]. The progression of disease begins within about 10–15 years in 50% of patients [1]. About 50% of patients with multiple sclerosis die of the disease or related problems such as suicide, severe falls, accidents, and a culmination of psychological issues [6,7]. Symptoms such as incontinence, cognitive impairment, bedsores, fatigue, and pain are the common symptoms in the severe stage of multiple sclerosis [1]. Similarly, these symptoms increase the social, emotional, and physical burden in this cohort [8], and they also affect carers at home. It is recommended to engage the specialists of palliative care in neurology as well as in care for patients with multiple sclerosis [9,10]. Since multiple sclerosis affects young adults and is treated with limited medical treatment options, this disease poses a considerable economic and social burden and requires lifetime support and management [11,12]. In Saudi Arabia, the findings of the first nationwide registry of multiple sclerosis revealed that the projected prevalence of this disease is 40.40 per 100,000 people [5]. The projected prevalence among Saudi nationals was reported to be much higher (61.95 per 100,000 Saudi people). Moreover, multiple sclerosis is common among the educated, younger, and female population [5].

It is crucial to assess the satisfaction of patients with multiple sclerosis regarding medical services, treatment, and understanding about the disease and services that they receive at a healthcare setting. Similarly, the patients’ satisfaction could affect their participation in the treatment of multiple sclerosis, thereby improving clinical outcomes. Further, the practice of evaluating satisfaction may facilitate comprehension of the patients’ views and is one of the criteria of the quality of healthcare services provided at a healthcare setting [13,14]. Evidence regarding the satisfaction of patients with multiple sclerosis towards the provision of healthcare services in a tertiary care setting is limited. Previous study in Saudi Arabia primarily focused on assessing medication adherence and its association with treatment satisfaction [15]. Therefore, we aimed to determine the satisfaction of patients with multiple sclerosis with the provision of healthcare services in a tertiary care setting, and the factors affecting their satisfaction.

## 2. Methods

### 2.1. Study Design and Setting

A cross-sectional study was conducted from November 2022 to December 2022 at King Saud Medical City, Riyadh, Saudi Arabia.

### 2.2. Inclusion and Exclusion Criteria

Patients aged 18 years and above with a confirmed clinical diagnosis of any type of multiple sclerosis attending the outpatient clinic of King Saud Medical City were invited to participate in this study. We excluded patients without a confirmed clinical diagnosis of multiple sclerosis.

### 2.3. Data Collection

A CANHELP Lite27 questionnaire [16] was used, which includes twenty-one items divided into the following six areas: general satisfaction (one item), relationship with the doctors (three items), disease management (nine items), communication (three items) and decision-making (four items), and well-being/feeling of peace (one item). All items were evaluated on a five-point Likert scale ranging from 1 (totally dissatisfied) to 5 (totally satisfied). Domain scores range from 20 to 100. A higher number represents greater satisfaction with care in the domain area. The hard copy of the study questionnaire was distributed to patients who visited King Saud Medical City.

### 2.4. Study Variables

Demographic data and clinical characteristics were collected. Demographic data such as age, gender, occupational status, marital status, duration of multiple sclerosis (years), education, and children (yes or no) were collected. In addition, we collected responses to all 21 items included in the questionnaire.

### 2.5. Ethical Considerations

The study objectives were included on the first page of the questionnaire under the consent statement to provide general information to the study subjects. All respondents confirmed their willingness to participate voluntarily by reading statements about the purpose and consent. All data were collected anonymously and handled confidentially. Access to the password-protected Excel file was only granted to the research team. The study was conducted in accordance with the Declaration of Helsinki. We initiated this non-invasive study after obtaining approval from the Institutional Review Board of the King Saud Medical City (Approval number: H1R1-07-Nov22-01, Approval Date: 14 November 2022).

### 2.6. Sample Size and Statistical Analysis

Based on literature evidence, 14% of patients with multiple sclerosis were satisfied with the provision of healthcare services [14]. Given this proportion, a 95% confidence level, and 5% margin of error, a sample size of 184 was estimated using an online calculator [17].

Data analysis was carried out using SPSS software, version 21 (IBM Corp., Armonk, NY, USA). Descriptive and inferential statistics were used to report the data. The descriptive analysis was undertaken using mean and standard deviations for continuous variables and percentage for qualitative variables. Checking for data normality was carried out using the Shapiro–Wilk test (with *p*-value ≥ 0.05 indicating a normally distributed continuous variable). The correlations among the analyzed factors were assessed through Spearman’s correlation coefficient. The predictors influencing satisfaction will be tested through multiple linear regression analysis. For multiple linear regression analysis, using the entry method, the variable entry criterion was set to 0.25, i.e., any variables found to be significant on the single predictor level (*p* < 0.25) were entered into the multiple linear regression analysis to explore the variables that were significantly and independently associated with participants’ satisfaction score. A *p*-value of less than 0.05 was considered statistically significant.

## 3. Results

### 3.1. Sociodemographic Characteristics of the Participants

The current study included 300 patients diagnosed with multiple sclerosis, who were receiving care at the outpatient clinic of King Saud Medical City. The sociodemographic characteristics of the patients, including age, duration of multiple sclerosis, gender, marital status, educational level, employment status, and children, are presented in Table 1. The findings revealed a predominantly female sample (80%), with a mean age of 31.6 ± 7.4 years and a mean duration of multiple sclerosis of 5.22 ± 3.69 years. The majority of participants were either single (39.0%) or married (37.3%), while smaller proportions were divorced (22.3%) or widowed (1.3%). Regarding parental status, 43.7% of participants reported having children, while 56.3% reported having no children. In terms of educational level, the largest proportion of participants had completed high school (55.3%), followed by those with a bachelor’s degree (25.7%), middle school education (16.3%), and postgraduate degree (2.7%). Approximately half of the participants were employed (52.7%), while the remaining half were unemployed (47.3%).

### 3.2. Evaluation of Patients’ Satisfaction with Healthcare Services

Table 2 presents the assessment of patients’ satisfaction with multiple sclerosis healthcare services across various domains using the CANHELP Lite questionnaire. The overall satisfaction score was 63.59 ± 14.54, indicating a moderate level of satisfaction among patients with multiple sclerosis regarding healthcare services. Patients exhibited the highest satisfaction levels with the general aspects of healthcare services, scoring a mean of 72.00 ± 16.46, followed closely by the domains of relationship with doctors (68.58 ± 14.88) and communication (67.72 ± 17.60). Conversely, satisfaction levels were comparatively lower for decision-making (64.98 ± 18.37), illness management (59.60 ± 16.31), and feeling at peace (58.17 ± 25.46).

### 3.3. Evaluation of Factors Affecting Patients’ Satisfaction with Multiple Sclerosis Healthcare Services

Correlation analysis was employed to examine the association between patients’ satisfaction and both their age and the duration of multiple sclerosis (Table 3). The findings indicated the lack of a significant relationship between age and patients’ satisfaction scores (*p*-value > 0.05)**.** However, the results indicated a significant negative correlation between duration of multiple sclerosis and all the domains of satisfaction among the patients with a correlation coefficient ranging from −0.290 to −0.206 (*p*-value < 0.01).

Furthermore, independent sample *t*-test and ANOVA were used to test the differences in patients’ satisfaction scores with respect to their gender, marital status, educational level, employment status, and parental status (Table 4). The results revealed significantly higher satisfaction among males in the domains of relationship with doctors and illness management. With respect to marital status, the results indicated higher satisfaction scores among married patients compared to singles in overall satisfaction and all satisfaction domains except for the feeling at peace domain.

In addition, the results showed significant differences in overall satisfaction and all satisfaction domains with respect to patients’ educational level. The results indicated a significant increase in general satisfaction with higher educational levels. On the other hand, the results indicated a significantly higher overall satisfaction and other satisfaction domains among patients with bachelor’s degree or higher compared to patients with middle and high school. With respect to employment status, the results revealed significantly higher general satisfaction among employed patients; however, the results showed no significant differences in overall satisfaction and other satisfaction domains with respect to patients’ employment status. Finally, the results suggested no significant differences in overall satisfaction and all its domains between patients with and without children.

### 3.4. Predictors of Satisfaction with Healthcare Services Among Patients

To investigate the predictors influencing satisfaction, multiple linear regression analysis was employed with satisfaction scores as the dependent variable and age, duration of multiple sclerosis, gender, marital status, educational level, employment status, and parental status each as independent variables (Table 5). The results revealed significant positive influence of patients’ age on general satisfaction, decision-making, communication, and overall satisfaction. In addition, the results indicated a significant negative influence of duration of multiple sclerosis on overall satisfaction and all its domains. With respect to patients’ gender, the results showed that females had significantly lower satisfaction in relationship with the doctors and illness management. In terms of marital status, the results showed a significant negative influence among divorced/widowed on general satisfaction compared to single patients. However, the results revealed significantly higher overall satisfaction and all satisfaction domains (except for feeling at peace) among married and divorced/widowed compared to single patients.

In terms of educational level, the results revealed a significantly higher overall satisfaction and all its domains among patients with bachelor’s degree or higher compared to patients with middle school. In addition, the results showed higher general satisfaction and illness management among high school compared to middle school patients. The results indicated no significant influence of employment status on overall satisfaction and all its domains; however, the results showed significantly lower illness management, communication, decision-making, and overall satisfaction among patients with children.

## 4. Discussion

According to the present study young adult females are more likely to experience MS. Similarly, evidence posits that females are four times more affected by MS than males, with a late onset and worse prognosis [18]. In addition, a study conducted by AlJumah et al. [5] suggested a higher prevalence of MS among females than males by 2:1. Ysrraelit and Correale [19] examined the effects of sex hormones on immune function and MS. The findings of the study suggested earlier disease onset and frequent relapse among females, while faster disease progression and worse outcomes are found in males.

In the present study, the overall level of satisfaction of MS patients was found to be moderate. Likewise, a study conducted by Albahrani et al. [20] on patients in primary healthcare centers in Saudi Arabia showed moderate satisfaction among patients. The healthcare system of Saudi Arabia has made significant progress; however, the system still needs improvements in some areas such as the workforce, preventive care, and health disparities. Thus, achieving these goals might result in a sustainable healthcare system [21]. In the present study, patients with MS showed satisfaction with general aspects of care, doctor relationships, and communication. However, the study participants were less satisfied with decision-making and illness management. It is clear that the availability of high-quality services such as good communication and information impacts patients’ overall satisfaction [22]. Moreover, participating in shared decision-making leads to improved satisfaction and lower decisional conflict among patients with MS [23].

The findings of the study showed a significant negative relationship between disease duration and satisfaction across all domains including relationship with a doctor, illness management, communication, decision-making, feeling at peace, and overall satisfaction. However, the literature suggests that as patients become accustomed to receiving treatment, their expectations are reduced which leads to a higher level of satisfaction [24]. Additionally, a study conducted by Donahue et al. [25] found satisfaction among patients who had longer relationships with their physicians. This indicates that the patients and the physicians prefer continuity [26]. According to Karampampa et al. [27], disease progression and relapse decreases the quality of life among patients with MS. Thus, based on the current study’s findings, it is recommended to adopt a holistic approach, addressing physical symptoms as well as socioemotional aspects of their well-being.

Regression analysis findings showed no significant role of age and patient satisfaction. The findings of Bjertnaes et al. [28] also showed a non-significant role of age in predicting overall patient satisfaction. The current study’s findings suggested increased satisfaction among patients with higher education and marital status but mixed findings for employment status and having children. Aljarallah et al. [24] conducted a study to examine patients’ satisfaction in tertiary care facilities in Riyadh Saudi Arabia. Findings indicated higher satisfaction among patients who are employed, academically certified, and married in comparison to unemployed, less qualified, and single. Thus, it can be assumed that patients with higher education may know about their health condition and understand treatment options, resulting in increased satisfaction. Moreover, married patients may have social support which could lead to satisfaction with healthcare services [29].

### 4.1. Strengths and Limitations

The present study entails following strengths: Foremost, the present study aimed to examine the satisfaction of patients with MS with healthcare services at a tertiary care setting in Saudi Arabia. Secondly, the strength of the current study is the use of a validated tool, the CANHELP Lite questionnaire to examine patients’ satisfaction with healthcare services. Moreover, variables such as demographic and clinical provide a comprehensive analysis of factors associated with patient satisfaction.

Despite strengths, the present study also entails some limitations, which are as follows: Firstly, the inclusion of study participants from King Saud Medical City, Saudi Arabia potentially leads to selection bias and limits the generalizability of the study findings to other healthcare settings. Secondly, the cross-sectional design only investigates the relationship between study variables and limits the causal relationship between variables. Similarly, the results should be understood as being indicative of associations rather than implying any causal relationships.

### 4.2. Implications for Clinical Practice and Future Research

Based on the present study’s findings, healthcare providers should tailor interventions to address the lower satisfaction with the domains of healthcare settings such as illness management and decision-making. Healthcare providers should prioritize long-term care involving symptom management and improving coping strategies with chronic illness. Moreover, future research should emphasize evaluating intervention studies that aim to improve the satisfaction of patients with MS. Additionally, longitudinal studies could be effective in tracking changes in satisfaction levels with care over time and exploring factors associated with the changes. Lastly, the inclusion of additional variables such as socioeconomic status and cultural factors may provide comprehensive information about the determinants of patient satisfaction.

## 5. Conclusions

This research uncovered significant insights regarding MS patient satisfaction within healthcare services. Despite moderate satisfaction levels overall, specific interventions are necessary to address shortcomings in decision-making and illness management. The negative correlation between disease duration and satisfaction across all domains underscores the evolving needs of MS patients over time. Future research could examine the effectiveness of illness management programs in improving MS patient satisfaction.

## Figures and Tables

**Table 1:** Sociodemographic Characteristics of the Participants.

Characteristics	n	%
Age *	31.60 ± 7.40 (18–63)	
Duration of multiple sclerosis *	5.22 ± 3.69 (1–26)	
Gender		
Male	60	20.0%
Female	240	80.0%
Marital Status		
Single	117	39.0%
Married	112	37.3%
Divorced	67	22.3%
Widow	4	1.3%
Educational Level		
Middle School	49	16.3%
High School	166	55.3%
Bachelor’s Degree	77	25.7%
Postgraduate Degree	8	2.7%
Employment Status		
Employed	158	52.7%
Unemployed	142	47.3%
Children		
Yes	131	43.7%
No	169	56.3%

* Mean ± SD (range).

**Table 2:** Patients’ satisfaction with multiple sclerosis healthcare services.

Domains	Mean	SD	Min.	Max.
General Satisfaction	72.00	16.46	25.0	100.0
Relationship with the Doctors	68.58	14.88	25.0	100.0
Illness Management	59.60	16.31	30.6	100.0
Communication	67.72	17.60	25.0	100.0
Decision-Making	64.98	18.37	25.0	100.0
Feeling at Peace	58.17	25.46	25.0	100.0
Overall Satisfaction	63.59	14.54	33.3	100.0

**Table 3:** Correlations between satisfactions with healthcare services and patients’ age and duration of multiple sclerosis.

Factors Affecting Patients’ Satisfaction	Age	Duration of Multiple Sclerosis
General Satisfaction	0.012	−0.237 *
Relationship with the Doctors	−0.008	−0.290 *
Illness Management	0.032	−0.278 *
Communication	0.059	−0.206 *
Decision-Making	0.046	−0.217 *
Feeling at Peace	−0.031	−0.227 *
Overall Satisfaction	0.056	−0.248 *

* Significant at 0.01 level.

**Table 4:** Differences in patients’ satisfaction with respect to demographic characteristics.

Demographics	General Satisfaction	Relationship with the Doctors	Illness Management	Communication	Decision-Making	Feeling at Peace	Overall Satisfaction
Gender							
Male	75.0 ± 15.3	73.2 ± 13.2	64.4 ± 17.5	70.7 ± 16.4	66.2 ± 17.5	57.5 ± 26.2	67.0 ± 14.9
Female	71.3 ± 16.7	67.4 ± 15.1	58.4 ± 15.8	67.0 ± 17.9	64.7 ± 18.6	58.3 ± 25.3	62.7 ± 14.4
*p*-value	0.115	0.007	0.011	0.144	0.289	0.821	0.04
Marital Status							
Single	72.7 ^a^ ± 17.1	66.0 ^a^ ± 14.3	55.6 ^a^ ± 15.6	64.7 ^a^ ± 18.2	60.7 ^a^ ± 19.1	57.3 ± 26.1	60.3 ^a^ ± 14.1
Married	75.5 ^a^ ± 13.0	71.1 ^b^ ± 14.8	63.2 ^b^ ± 16.1	70.4 ^b^ ± 16.4	69.8 ^b^ ± 16.5	61.4 ± 23.9	67.1 ^b^ ± 13.9
Divorced/Widowed	65.5 ^b^ ± 18.6	68.8 ^a,b^ ± 15.5	60.5 ^b^ ± 16.6	68.5 ^a,b^ ± 18.0	64.4 ^a,b^ ± 18.4	54.6 ± 26.5	63.6 ^a,b^ ± 15.1
*p*-value	<0.001	0.034	0.002	0.044	<0.001	0.188	0.002
Educational Level							
Middle School	64.3 ^a^ ± 15.3	65.1 ^a^ ± 14.0	53.9 ^a^ ± 14.5	63.8 ^a^ ± 18.1	61.4 ^a^ ± 17.3	53.6 ^a^ ± 21.7	58.8 ^a^ ± 12.7
High School	71.4 ^b^ ± 14.9	66.3 ^a^ ± 13.3	57.4 ^a^ ± 14.2	65.0 ^a^ ± 16.3	62.4 ^a^ ± 17.3	54.4 ^a^ ± 24.3	61.2 ^a^ ± 12.4
Bachelor’s degree or higher	77.7 ^c^ ± 18.1	75.0 ^b^ ± 16.4	67.3 ^b^ ± 18.5	75.3 ^b^ ± 17.6	72.1 ^b^ ± 19.2	68.2 ^b^ ± 27.1	71.0 ^b^ ± 16.7
*p*-value	<0.001	<0.001	<0.001	<0.001	<0.001	<0.001	<0.001
Employment Status							
Employed	73.9 ± 15.3	69.5 ± 13.9	59.3 ± 15.9	67.1 ± 17.5	64.8 ± 17.4	59.5 ± 25.0	63.6 ± 13.9
Unemployed	69.9 ± 17.5	67.6 ± 15.9	59.9 ± 16.8	68.4 ± 17.7	65.1 ± 19.5	56.7 ± 25.9	63.5 ± 15.3
*p*-value	0.037	0.253	0.751	0.512	0.885	0.342	0.954
Children							
Yes	72.1 ± 14.8	68.8 ± 14.3	59.9 ± 14.9	67.4 ± 17.2	65.3 ± 16.7	56.9 ± 24.4	63.7 ± 13.3
No	71.9 ± 17.7	68.4 ± 15.4	59.3 ± 17.4	68 ± 17.9	64.7 ± 19.6	59.2 ± 26.3	63.5 ± 15.5
*p*-value	0.897	0.801	0.749	0.758	0.777	0.438	0.883

^a^: Statistically significant; ^b^: Statistically significant; ^c^: Statistically significant.

**Table 5:** Predictors of satisfaction with healthcare services.

Independent Variables	General Satisfaction		Relationship with the Doctors		Illness Management		Communication		Decision-Making		Feeling at Peace		Overall Satisfaction	
	β	*p*-Value	β	*p*-Value	β	*p*-Value	β	*p*-Value	β	*p*-Value	β	*p*-Value	β	*p*-Value
Age	0.178	0.024	0.144	0.059	0.131	0.074	0.224	0.005	0.190	0.016	0.129	0.113	0.189	0.011
Duration of multiple sclerosis	−0.270	<0.001	−0.390	<0.001	−0.378	<0.001	−0.258	<0.001	−0.224	0.002	−0.248	0.001	−0.372	<0.001
Female	−0.061	0.300	−0.144	0.012	−0.154	0.006	−0.067	0.260	0.002	0.977	0.031	0.610	−0.107	0.054
Married	0.066	0.441	0.258	0.002	0.372	<0.001	0.260	0.003	0.372	<0.001	0.153	0.083	0.367	<0.001
Divorced/Widowed	−0.178	0.017	0.163	0.024	0.253	<0.001	0.166	0.026	0.186	0.013	0.022	0.777	0.211	0.003
Bachelor’s degree or higher	0.287	<0.001	0.209	0.008	0.328	<0.001	0.295	<0.001	0.266	0.001	0.189	0.024	0.335	<0.001
High School	0.171	0.034	0.031	0.687	0.151	0.045	0.088	0.274	0.090	0.264	−0.016	0.843	0.122	0.106
Employed	0.042	0.469	0.021	0.707	−0.078	0.154	−0.097	0.098	−0.039	0.503	0.045	0.453	−0.055	0.320
Children	0.027	0.732	−0.111	0.144	−0.158	0.032	−0.195	0.013	−0.211	0.008	−0.105	0.197	−0.184	0.013
Model Sum.	<0.001		<0.001		<0.001		<0.001		<0.001		<0.001		<0.001	

## Data Availability

All data are included in the manuscript.
